# The relationship between mixed exposure to blood metal and serum neurofilament light chain levels in the general U.S. population: an unsupervised clustering approach

**DOI:** 10.3389/fpubh.2025.1516879

**Published:** 2025-07-30

**Authors:** Jiyu Nie, Lin Wen, Zhentian Lai, Chuhang Lin, Haiyin Li, Jing Zhang, Shen Xie, Xiaosong Ben, Chunxia Jing

**Affiliations:** ^1^Department of Public Health and Preventive Medicine, School of Medicine, Jinan University, Guangzhou, China; ^2^Guangdong Key Laboratory of Environmental Exposure and Health, Jinan University, Guangzhou, China; ^3^Department of Medical Records, Guangdong Provincial People’s Hospital (Guangdong Academy of Medical Sciences), Southern Medical University, Guangzhou, China; ^4^Department of Thoracic Surgery, Guangdong Provincial People's Hospital, Guangdong Academy of Medical Sciences, Guangzhou, China

**Keywords:** metal exposure, serum neurofilament light chain, unsupervised machine learning, BKMR, G-computation

## Abstract

**Background:**

Serum neurofilament light chain (sNfL) has demonstrate significant clinical value in quantifying neuronal injury. Concurrently, extensive evidence has linked metal exposure to neurotoxic effects. However, the potential association between metal exposure and circulating sNfL levels remains uninvestigated in population-based study.

**Objective:**

We applied a novel unsupervised clustering method (k-medoids) incorporating blood metals concentrations to stratify the general U. S. population into different exposure profiles to investigate the association between metal exposure and sNfL levels.

**Methods:**

We analyzed data from the 2013–2014 NHANES cycle, and 513 participants were included in this study. Multivariate regression model, Bayesian kernel regression (BKMR) and quantile g-computation (QGC) were used to assess the relationship between individual and mixed metal exposure and sNfL.

**Results:**

Multivariate regression revealed a significant positive association between blood cadmium concentrations and elevated sNfL levels in the overall population (β = 0.115, 95%CI: 0.039–0.190, *p* = 0.003). Through exposure pattern recognition using unsupervised k-medoids clustering, participants were stratified into distinct exposure subgroups: a high-exposure cluster (*n* = 326) and a low-exposure (*n* = 187) reference group. BKMR modeling within the high-exposure group identified cadmium as the dominant contributor to sNfL elevation, with stronger effects in male participants (β = 0.201, 95%CI: 0.087–0.315) and individuals with BMI > 25 kg/m^2^ (β = 0.178, 95%CI: 0.062–0.294).

**Conclusion:**

This study provides systematic evidence that blood cadmium concentration can be used as the predominant driver of early neuronal injury, as objectively quantified through sNfL biomarker.

## 1 Introduction

Neurofilament (Nf) is a major structural protein involved in maintaining neuronal specificity. It comprises four subunits, including the neurofilament light chain (NfL) (1). When axonal damage or neuronal degeneration occurs, NfL is released in large quantities. The rupture of the axonal membrane leads to the release of NfL into the interstitial fluid, which eventually reaches the cerebrospinal fluid (CSF) and blood (2). This makes it possible to detect and measure NfL levels in the CSF and blood, serving as a biomarker for axonal damage and neuronal degeneration. NfL has been associated with various neurological diseases such as amyotrophic lateral sclerosis (3), frontotemporal dementia (4), and progressive supranuclear palsy (5). In multiple sclerosis, NfL levels have been shown to be a crucial predictor of disease progression and cerebrospinal atrophy (6). Notably, serum NfL (sNfL) levels correlate with disease severity and progression and have prognostic and diagnostic value in clinical practice. For example, different subtypes of frontotemporal dementia are associated with different levels of NfL (7). In Alzheimer’s disease, elevated levels of sNfL often accompany network uncoupling and cognitive decline (8).

Despite technological advances (9, 10), escalating heavy metals contamination from accelerated industrialization, climate change and environmental pollution drive widespread daily exposure exceeding global safety thresholds, particularly in developing countries (11). Exposure to environmental toxicants, including metal pollution, has been shown to have detrimental effects on neurological function (12). Heavy metals such as cadmium, lead, manganese, copper, and mercury are known neurotoxicants and pose significant risks to neuronal function (13, 14). Animal studies have shown that subacute exposure to dissolved cadmium and cadmium nanoparticles can lead to apparent neurological impairment in rats (15). Cadmium-induced neurodegeneration and cognitive impairment are associated with oxidative stress, which reduces neuronal differentiation and axon genesis, resulting in nerve damage and neuronal cell death (16). Low-level lead poisoning has been linked to reduced subcortical brain structures and cognitive deficits, as evidenced by a cohort study of 9–10-year-old participants in the United States (17). Manganese, as a transition metal, can participate in redox reactions, and manganese-induced oxidative damage has been linked to neuronal degeneration (18). Zinc can provide neuroprotection in spinal contusion model by modulating the NLRP3 inflammasome through autophagy and ubiquitination mechanisms (19). While existing occupational cohort studies and animal models have elucidated the mechanisms of single-metal toxicity, critical knowledge gaps persist regarding mixed-metal exposure effects in general population. Current research face two fundamental challenges: (1) The inherent complexity of environmental exposure matrix, where real-world populations encounter concurrent multi-metal exposures, but most studies have focused on isolated contaminants, ignoring potential synergistic or additive effects. (2) Insufficient investigation of potential supra-additive or synergistic neurotoxic interaction. To address these critical knowledge gaps, we presented an innovative exposure framework that synergistically integrates unsupervised machine learning with advanced mixture exposure analytics [including BKMR and (QGC), using the National Health and Nutrition Examination Survey (NHANES 2013–2014)] data to quantify the neurotoxic effects of multi-metal co-exposure on sNfL levels.

## 2 Methods

### 2.1 Study population

The National Health and Nutrition Examination Survey (NHANES) is a research program to assess adult’s and children’s health and nutritional status in the United States. We used cross-sectional data from NHANES 2013–2014 cycles for our analysis. NHANES employed a complex multistage probability sampling strategy to select a sample of civilian, non-institutionalized individuals to enhance representativeness. The NCHS Research Ethics Review Committee carefully reviewed the NHANES protocol, and each participant provided written consent before study participation. These rigorous ethical protocols and procedures uphold the highest scientific standards of integrity and safeguard the rights and welfare of research participants. Our study investigated the relationship between mixed metal exposure and sNfL levels in a diverse and nationally representative sample of the U. S. population.

**Figure 1:**
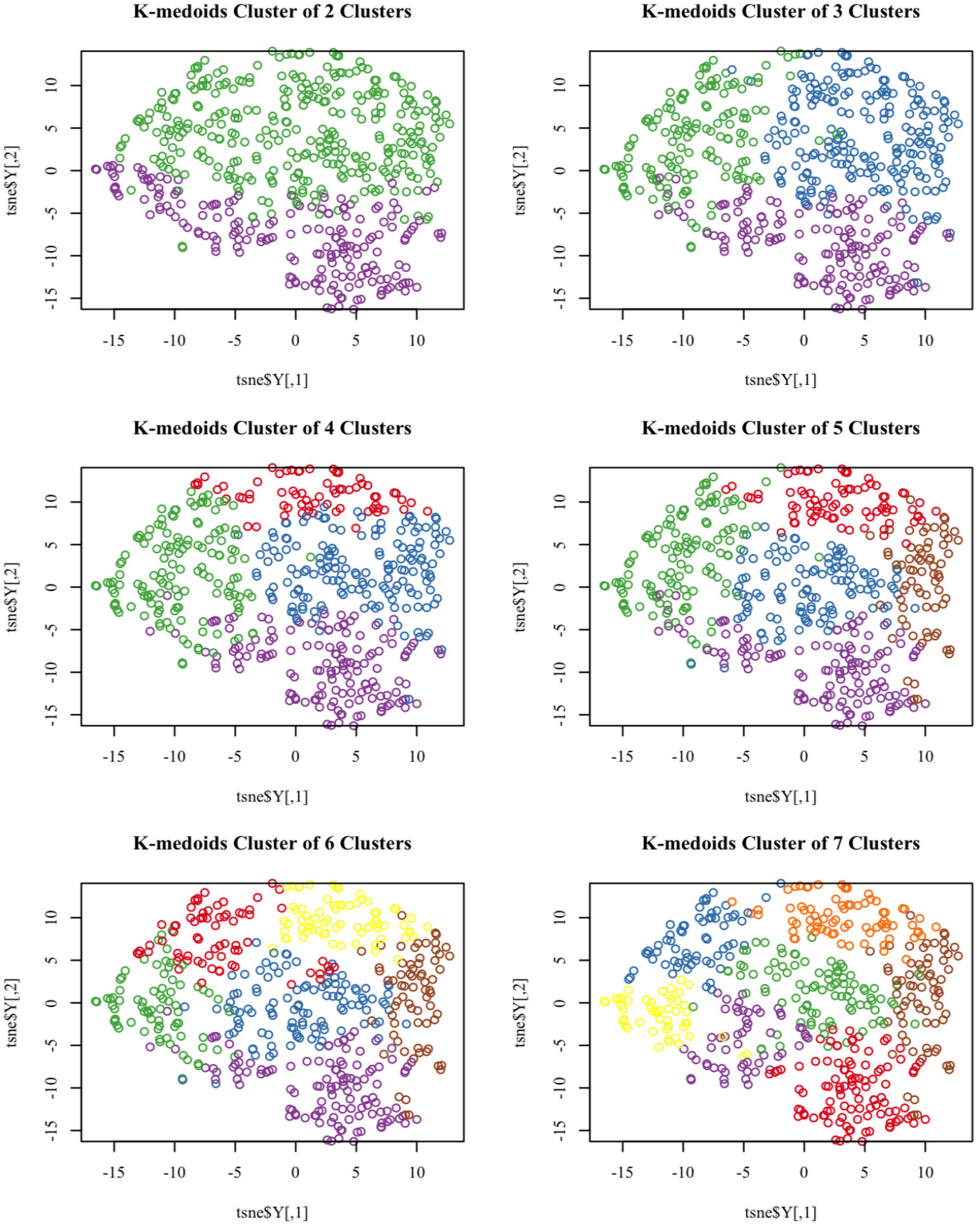
T-SNE visualized k-medoids from 513 NHANES subjects based on metals in blood, with the number of clusters set from 2 to 7, respectively.

The data from NHANES 2013–2014 was analyzed, and the 10,175 participants were screened. We merged the databases based on the unique identity of the survey subjects. After merging the databases, we excluded 9,662 who had missing data in drinking, smoking, neurofilaments light chain data and blood metal measurements. Finally, 513 survey subjects were included in the study (Figure 1).

### 2.2 Measurement of serum sNfL

Detection of sNfL involves using a highly sensitive immunoassay developed by Siemens Healthiness. This process utilizes acridine chemiluminescence and paramagnetic particles and runs on an existing high-throughput automation platform.

First, samples are incubated with antibodies labeled with AE and bind to the sNfL antigen. Then, paramagnetic particles coated with the captured antibody are added to the sample to form a complex that binds the antigen to the AE-labeled antibody and PMP. Unbound AE-labeled antibodies are then isolated and removed. Finally, acids and bases are added to induce chemiluminescence, and the luminescence is measured. This entire process is performed on a fully automated Attelica immunoassay system.

### 2.3 Measurement of blood metals

The blood samples were stored at −30°C until transported to the National Centre for Environmental Health for testing. The concentrations of lead (Pb), cadmium (Cd), total mercury (Hg), manganese (Mn), and selenium (Se) in whole blood were detected using inductively coupled plasma mass spectrometry. In addition, serum was collected and stored at −20°C for analysis of copper (Cu) and zinc (Zn) using inductively coupled plasma Dynamic Response cell mass spectrometry (ICP-DRC-MS).

Detailed data declarations for blood heavy metal testing can be found at: https://wwwn.cdc.gov/Nchs/Nhanes/2017-2018/PBCD_J.htm-Analytic_Notes.

### 2.4 Covariates

The 15 covariates that may be associated with sNfL and blood metal were chosen based on previous research (20–22), including age, sex, race, educational status, marital status, family income to poverty ratio, BMI, smoking, alcohol consumption, physical activity, urinary creatinine level, hypertension, depression, cardiovascular disease, and hyperuricemia. Smoking was defined by a self-report questionnaire and categorized as never smoked (Participants answering “No” to the question, “Smoked at least 100 cigarettes in life”), ever smoked (Participants answering “Yes” to the question, “Smoked at least 100 cigarettes in life” and answering “Not at all” to the question,” Do you now smoke cigarettes?”) and current smoker (Participants answering “Yes” to the question, “Smoked at least 100 cigarettes in life” and answering “Every day” to the question,” Do you now smoke cigarettes?” or Participants answering “Yes” to the question, “Smoked at least 100 cigarettes in life” and answering “Some days” to the question,” Do you now smoke cigarettes?”). Hypertension will be defined based on participants’ self-report (answer “yes” to the question “ever been told I have hypertension”) or report current use of anti-hypertensive medication (participants answer “yes” to the question “taking a prescription for hypertension”). If participants reported drinking alcohol (participants answered “Yes” to the question “Have you had at least 12 drinks per year?” by responding “yes” to the question), then they will be defined as having consumed alcohol. The definition of physical activity will be based on participants’ self-reports (answering “yes” to the question, “Do you engage in at least 10 min of continuous exercise during the week, or do you engage in any moderate-intensity exercise that causes slight increases in breathing or heart rate for at least 10 min continuously during the week?”). Hyperuricemia is defined as (LBXSUA ≥ 7 in males and LBXSUA ≥ 6 in females). The definition of depression will be based on the quantitative score obtained from a depression screening tool. A score of 10 or higher will be defined as depression or depressive tendencies. The definition of cardiovascular disease will be based on participants’ self-reports (answering “yes” to the question “Have you ever been told you have congestive heart failure/coronary heart disease/angina/heart disease/stroke?”) and will be defined as having cardiovascular disease.

### 2.5 Unsupervised cluster analysis process

We employ the unsupervised clustering method K-medoids to group the research objects (23, 24). The K-medoids algorithm differs from the K-means algorithm in that it uses the most central objects in the cluster, known as the medoids, as reference points. This algorithm shares similar procedural steps with K-means but serves as an enhancement and optimization of the K-means method. Notably, K-medoids must select the sample point each time it chooses the center of mass, while K-means can select the center of mass from points other than the sample point, akin to the distinction between the median and the mean. The focus of K-medoids lies in the selection of centroids.

The criterion function for selecting the cluster centroid is to minimize the sum of distances from all other points in the current cluster to that centroid, necessitating the traversal of all points in the cluster (25). The steps of the K-medoids algorithm are as follows:

(1) Arbitrarily select k points to serve as the medoids. (2) Based on the principle of proximity to the medoids, assign the remaining points to the class represented by the best current medoids. (3) Calculate the criterion function corresponding to each member point within each class and select the points with the smallest criterion function as the new medoids. (4) Repeat the process of steps 2–3 until the medoid points no longer change or the maximum number of iterations is reached.

### 2.6 Statistical analysis

Continuous variables were presented as mean and standard deviation (Mean ± SD), while categorical variables were expressed as the number of cases (n) and percentage (%). To ensure normal distribution, the blood metal levels were log-transformed. The relationship between blood metals and sNfL levels was analyzed using multiple linear regression. Each blood metal was treated as a separate predictor in the regression models to evaluate their relationship with sNfL levels in a representative US population. Two models (Model 1 and Model 2) were used to ensure model stability. Model 1 did not adjust for any covariates, while Model 2 included all covariates. We then used the unsupervised clustering method, k-medoids, to divide the population into subgroups based on the concentration of mixed metal in the samples, thereby dividing the high-exposure and low-exposure groups. Due to the limitations of linear regression methods in dealing with high-dimensional data and nonlinear exposure-outcome relationships, Bayesian kernel-machine regression (BKMR) and quantile g-computation (QG-C) methods were used to estimate the effects of mixed-metal exposures on sNfL levels in different exposure groups. The BKMR models generated posterior incorporation probabilities (PIPs) ranging from 0 to 1, indicating the relative contribution of each blood metal to sNfL levels. The QGC model uses quantum g-computing to estimate the weights of *ψ* and assess the degree of violation of the directional homogeneity assumption. In addition, the model can also be used to assess the contributions of various environmental compounds (26).

This study demonstrated that the technique can reliably infer exposure effects across the mixture and determine the contribution of individual components without assuming directional homogeneity. All statistical analyses were performed using STATA (version 15.1) and R software (version 4.2.2).

## 3 Results

### 3.1 Unsupervised clustering and population classification

Based on the levels of multiple heavy metals in the blood, the K-medoids unsupervised clustering algorithm was employed to identify high and low exposure groups within the study population. T-SNE is a nonlinear dimensionality reduction technique that can embed high-dimensional data into two or three-dimensional space and is also utilized as a machine learning algorithm for cluster visualization (27).

**Figure 2:**
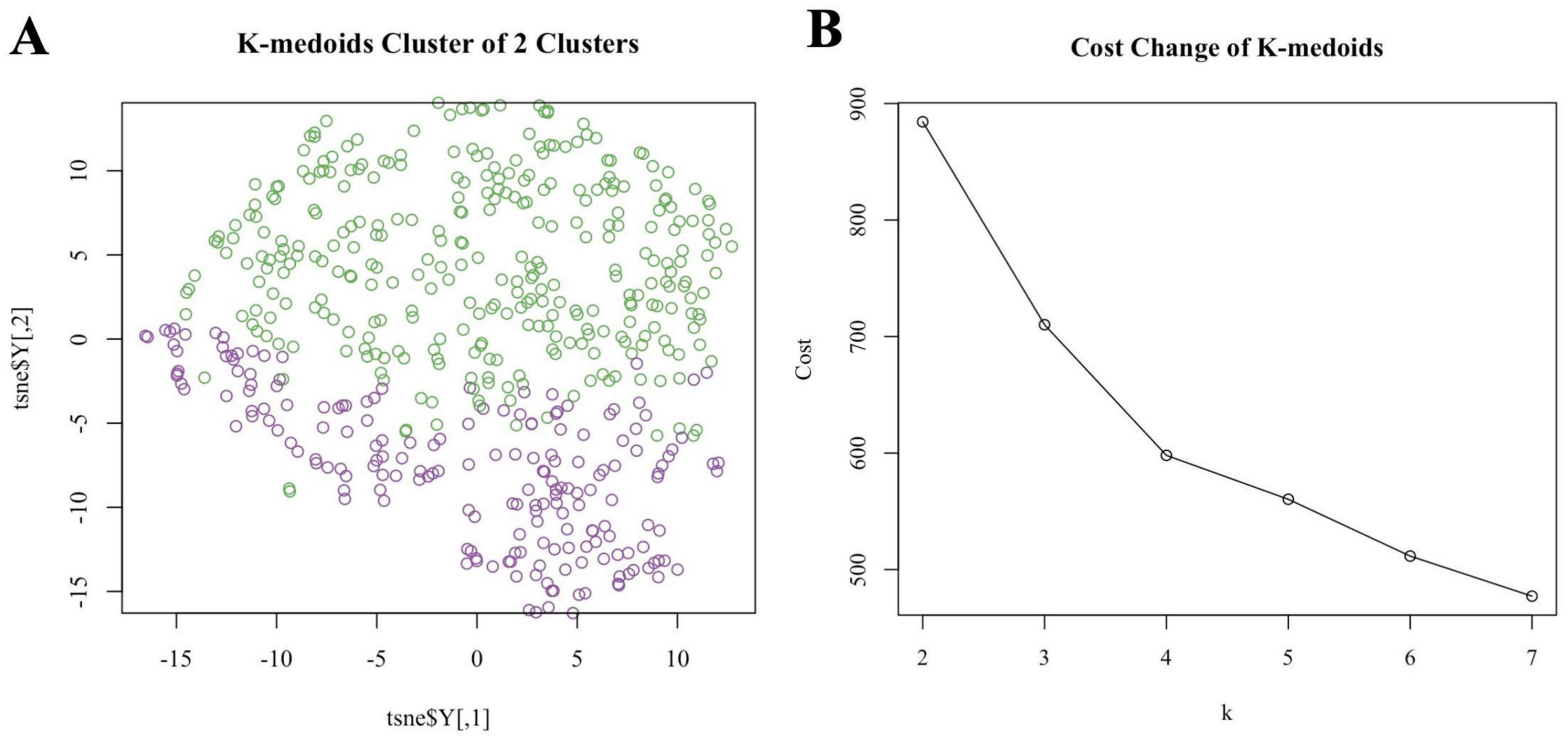
**(A)** T-SNE visualized k-medoids from 513 NHANES subjects based on metals in blood, with the number of clusters set to 2 and the upper half high exposure and the lower part low exposure. **(B)** The process for determining the optimal number of clusters for k-medoids using mean contour width as a metric and the elbow method as a criterion is described below.

Figure 1 demonstrates the T-SNE visualization of k-medoids from 513 NHANES subjects based on the metal content of their blood, with the number of clusters set from 2 to 7, respectively. And the results of T-SNE, which are shown in Figure 2, demonstrate that 326 subjects were classified into the high exposure group, while 187 subjects were classified into the low exposure group based on the content of heavy metals in their blood. Additional information on the centers of mass for the two subgroups based on the metal mixtures in blood or urine can be found in Table 1.

**Table 1:** The central mass of the two exposure groups after logarithmic transformations based on the metal mixtures in the blood.

Metals	Low-exposure (group 1)	High-exposure (group 2)
Blood Pb	−0.397	0.210
Blood Cd	−0.253	1.631
Blood Hg	−0.386	0.002
Blood Mn	2.206	2.262
Blood Se	5.262	5.293
Serum Cu	4.749	4.777
Serum Zn	4.483	4.456

### 3.2 Demographic characteristics

K-medoids clustering classified participants into a high-exposure group (*n* = 326, 63.6%) and a low-exposure group (*n* = 187, 36.4%). T-SNE visualization confirmed clear separation between groups (Figure 2A). The high-exposure group exhibited elevated blood cadmium (1.63 vs. −0.25 log-transformed units) and lead (0.21 vs. −0.40) levels (Table 1). There were significant differences (*p* < 0.01) between the high and low exposure groups in terms of age distribution, race, marital status, BMI index, smoking and drinking, as well as the presence of cardiovascular and cerebrovascular diseases. Specifically, the high exposure group had a higher proportion of non-older adult individuals (90.5% vs. 76.5%) and non-smokers (70.6% vs. 20.9%) compared to the low exposure group. Table 2 presents the participants’ demographics and individual characteristics.

**Table 2:** Demographic and individual characteristics of the study population.

	Low exposure (*N* = 187)	High exposure (*N* = 326)	*p-*value
Age group [*n*(%)]			
20 ~ 60 years	127 (67.9%)	258 (79.1%)	0.006**
Over 60 years	60 (32.1%)	68 (20.9%)	
Sex [*n*(%)]			
Male	104 (55.6%)	167 (51.2%)	0.386
Female	83 (44.4%)	159 (48.8%)	
Race [*n*(%)]			
Mexican American	16 (8.6%)	63 (19.3%)	0.004**
Other Hispanic	14 (7.5%)	37 (11.3%)	
Non-Hispanic White	98 (52.4%)	150 (46.0%)	
Non-Hispanic Black	38 (20.3%)	47 (14.4%)	
Other race—including multi-racial	21 (11.2%)	29 (8.9%)	
Marital status [*n*(%)]			
Married/living with partner	97 (51.9%)	204 (62.6%)	<0.001***
Never married	35 (18.7%)	76 (23.3%)	
Widowed/divorced/separated	55 (29.4%)	46 (14.1%)	
Education level [*n*(%)]			
Under high school	43 (23.0%)	52 (16.0%)	0.011*
High school or equivalent	48 (25.7%)	63 (19.3%)	
Above high school	96 (51.3%)	211 (64.7%)	
Ratio of family income to poverty [*n*(%)]			
Poverty	53 (28.3%)	64 (19.6%)	0.031*
Above poverty	134 (71.7%)	262 (80.4%)	
Smoking [*n*(%)]			
Smoking	148 (79.1%)	96 (29.4%)	<0.001***
No smoking	39 (20.9%)	230 (70.6%)	
Drinking [*n*(%)]			
Less than 12 alcoholic beverages/1 year	143 (76.5%)	295 (90.5%)	<0.001***
12 alcoholic beverages/1 year or more	44 (23.5%)	31 (9.5%)	
Exercise [*n*(%)]			
Not exercising regularly	106 (56.7%)	151 (46.3%)	0.030*
Exercise regularly	81 (43.3%)	175 (53.7%)	
Hypertension [*n*(%)]			
Yes	76 (40.6%)	100 (30.7%)	0.028*
No	111 (59.4%)	226 (69.3%)	
Depression [*n*(%)]			
Yes	21 (11.2%)	22 (6.7%)	0.110
No	166 (88.8%)	304 (93.3%)	
Diabetes [*n*(%)]			
Healthy	165 (88.2%)	283 (86.8%)	0.742
Diabetes	22 (11.8%)	43 (13.2%)	
Cardiovascular disease [*n*(%)]			
Yes	27 (14.4%)	17 (5.2%)	<0.001***
No	160 (85.6%)	309 (94.8%)	
Nonalcoholic fatty liver disease [*n*(%)]			
Yes	94 (50.3%)	196 (60.1%)	0.038*
No	93 (49.7%)	130 (39.9%)	
Hyperuricemia [*n*(%)]			
Yes	42 (22.5%)	67 (20.6%)	0.692
No	145 (77.5%)	259 (79.4%)	
BMI index [*n*(%)]			
Mean (SD)	3.30 (0.238)	3.36 (0.223)	0.003**
Median [min, max]	3.29 [2.83, 4.14]	3.34 [2.90, 4.25]	
Urine creatinine [*n*(%)]			
Mean (SD)	4.52 (0.763)	4.55 (0.660)	0.74
Median [min, max]	4.60 [1.61, 6.30]	4.61 [2.40, 6.05]	

*P < 0.05; **P < 0.01; ***P < 0.001. *P*-t, *p*-value for trend.

**Figure 3:**
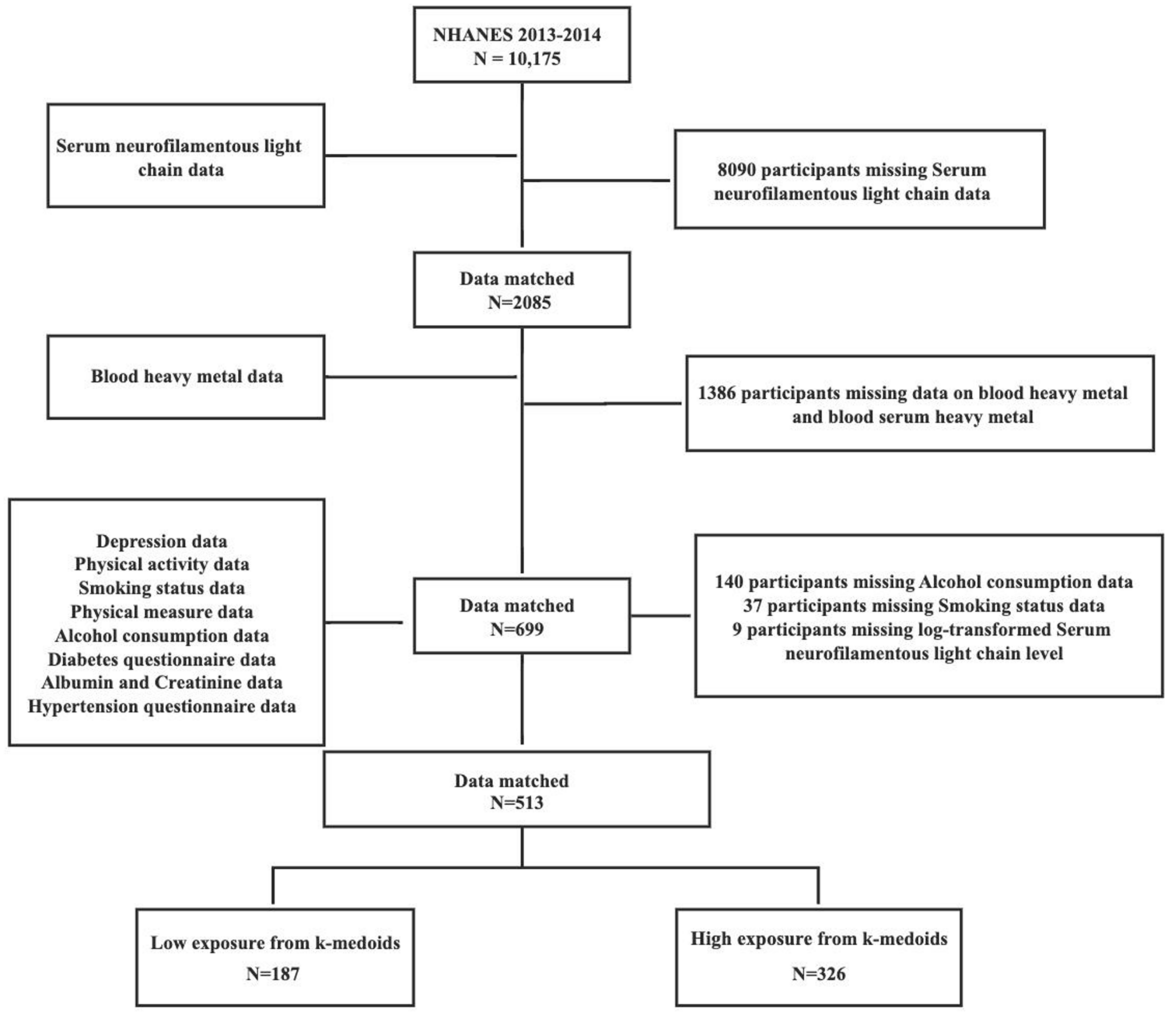
Flowchart for the selection of eligible participants.

To assess participants’ exposure to blood metals, we summarized the distribution and frequency of detection of these metals. Supplementary Table S1 presents the percent detectable, geometric mean, weighted mean, and percentage of detectable blood metals with detection levels greater than or equal to the LOD for the seven blood metals. At the same time, Supplementary Figure S1 shows the correlation heat maps of seven blood metals, indicating that the correlation between the most metals is not statistically significant. The participant screening process chart shows that we matched the survey subjects to the database based on their unique identifiers and ultimately included 513 survey subjects, which were then divided into a high exposure group (*n* = 326) and a low exposure group (*n* = 187) based on unsupervised clustering (Figure 3).

### 3.3 Associate between single metals and sNfL using multiple linear regression

**Figure 4:**
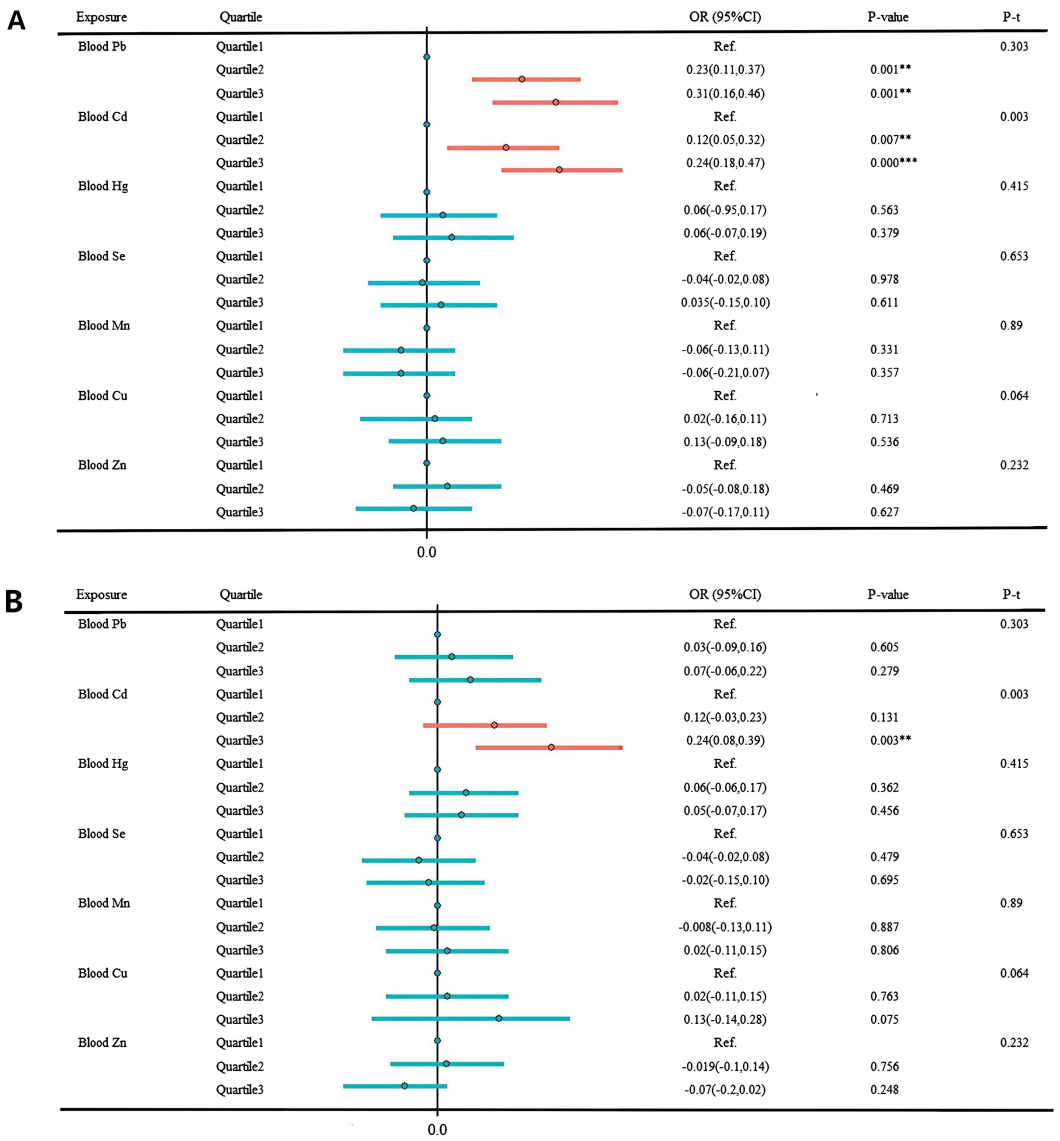
Multivariate regression results result of individual metals and sNfL. **(A)** Unadjusted model. **(B)** Adjusted for age group, sex, race, marital status, BMI index, smoking, drinking, exercise, education level, marital status, ratio of family income to poverty, urine creatinine, hypertension, depression, cardiovascular disease, and hyperuricemia. *p < 0.05; **p < 0.01; ***p < 0.001.

In Figure 4, the multivariate regression results examining the associations between individual blood metals and sNfL are presented. In the unadjusted model, significant correlations were found between both blood Cd and Pb levels and sNfL. Comparing the highest tertile concentration to the lowest tertile, each 1 standard deviation (SD) increment in blood Cd was associated with a significant increase of 0.159 pg./mL in sNfL (95% CI: 0.170–0.459, *p* = 0.000). Similarly, each 1 SD increase in blood Pb was associated with a significant increase of 0.154 pg./mL in sNfL (95% CI: 0.175–0.467, *p* = 0.000). After adjusting for all covariates, the statistical association between Pb and sNfL levels remained significant. The effect size per SD increase in blood Cd was 0.115 pg./mL (95%CI: 0.083–0.387, *p* = 0.003) when comparing to the highest tertile.

### 3.4 The effects of different exposure groups using the BKMR model

**Figure 5:**
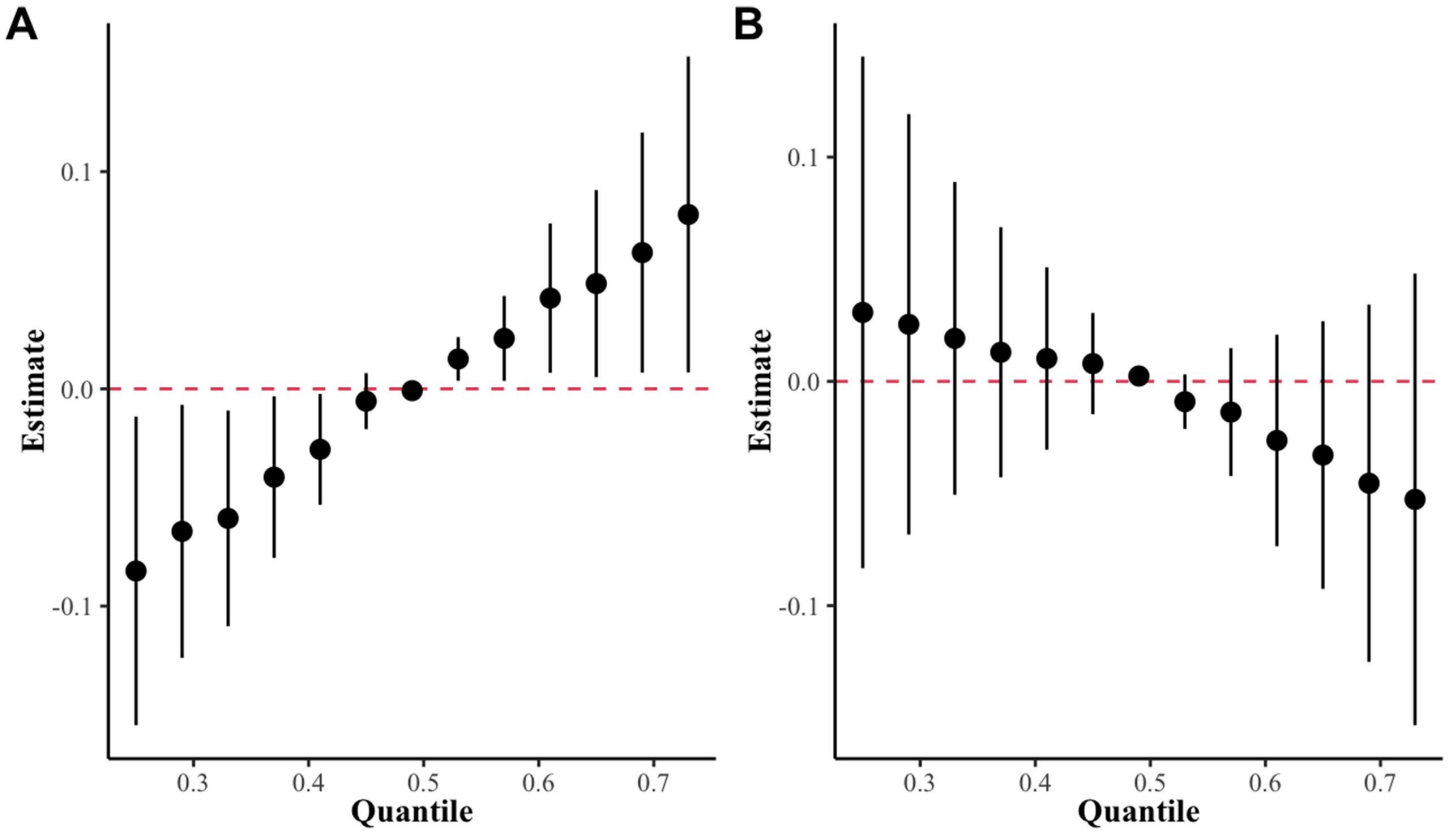
The overall effect of the metal mixture in the BKMR model. **(A)** High exposure group as determined by k-medoids clustering. **(B)** Low exposure group as determined by k-medoids clustering.

In this study, the BKMR model revealed a statistically significant positive correlation between co-exposure to the seven blood metals and sNfL levels when metal exposure was set at the 50th percentile in the high-exposure group. However, no such correlation was observed in the low-exposure group (Figure 5). Supplementary Table S2 provides a summary of groupPIP and condPIP for each metal, with the second group having the highest groupPIP (PIP = 0.955) and blood Cd showing the most significant contribution (condPIP = 1.000), suggesting it may play a crucial role in the association with sNfL.

**Figure 6:**
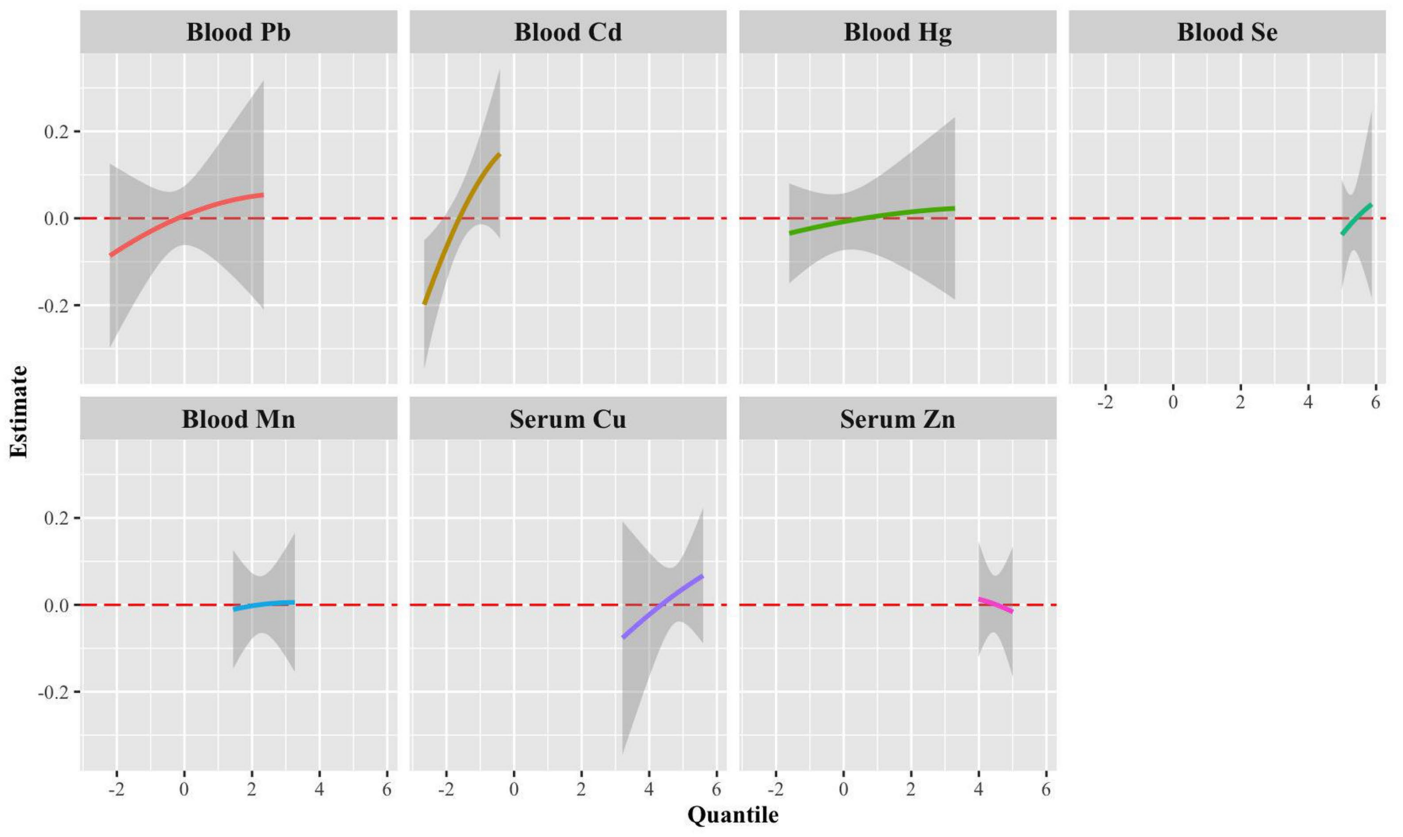
Univariate exposure-response functions (95% CrI) for a single metal associated with sNfL levels when other blood metals were fixed to the median.

Univariate exposure-response functions of single metals with sNfL levels showed significant positive dose–response curves for blood Pb, blood Cd, and blood Cu when we set the exposures to the other metals at the median value (Figure 6).

We also explore the interaction between blood metals using the BKMR package, fixing them at the 50th percentile and plotting the bivariate exposure-response functions for each metal at the 25th, 50th, and 75th percentiles. Results showed that the slope of the bivariate reaction function for any given blood metal remains constant across the different quantiles of other blood metals, suggesting no potential interaction between the various metals (Supplementary Figure S2).

The univariate effect plot in BKMR illustrates that, the exposure to Cd exhibits a statistically significant positive association with sNfL, when controlling other metal exposures at levels of 0.25, 0.5, and 0.75 content (Supplementary Figure S3).

### 3.5 Subgroup stratified and interaction analysis

**Figure 7:**
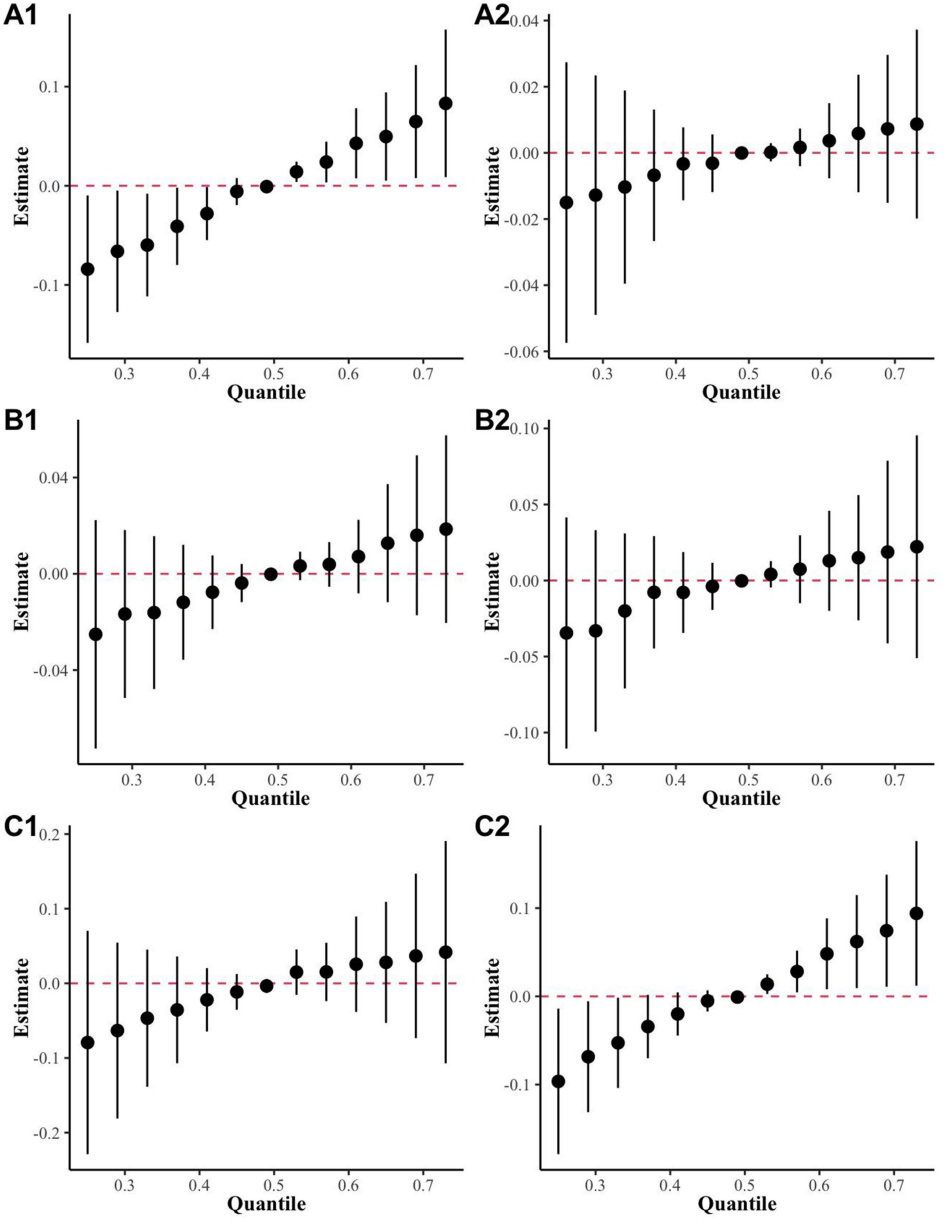
The BKMR results for mixed metal exposure and sNfL levels in different subgroups were stratified and analyzed in the highly exposed group. **(A)** Sex (**A1**: Male, **A2**: Female); **(B)** age (**B1**: over 60 years, **B2**: under 60 years); **(C)** BMI (**C1**: over 25 kg/m2, **C2**: under 25 kg/m^2^).

The results of multiple linear regressions in Supplementary Tables S3–S5 suggests that exposure to a mixture of metals may cause more nerve damage in male, and overweight groups. The stratified analysis by gender, age, and BMI based on the BKMR model identified significant positive trends in the male and overweight groups (Figure 7).

### 3.6 The effects of different exposure groups using QGC analysis

**Figure 8:**
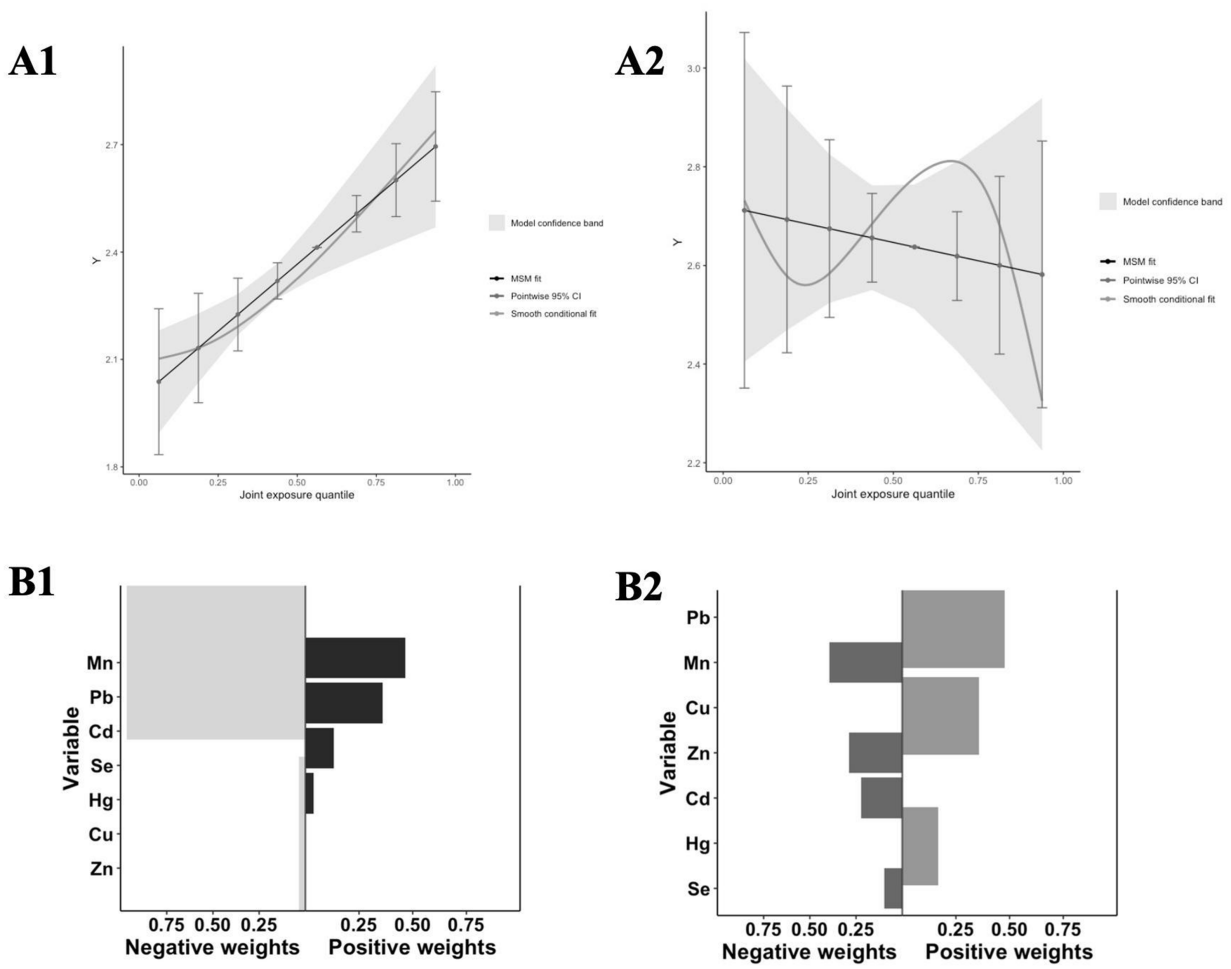
Analysis results of the QGC model. **(A)** The joint effects of mixed metal exposure on sNfL levels by QGC (**A1**: High exposure, **A2**: Low exposure); **(B)** Weight of each t metal with sNfL levels by QGC (**A1**: High exposure, **A2**: Low exposure).

The QGC model in Figure 8A1 revealed a significant positive correlation between mixed metal exposure and sNfL in the high exposure group (*p* < 0.001), while no statistical significance was observed in the low exposure group. This results was consistent with those of the BKMR model. Figure 8B displays the relative weights of each metal in relation to sNfL. In the high-exposure group, the negative weights of metals Mn, Zn, and sNfL were the largest, whereas the positive weights of metals Pb and Cd, along with sNfL, ranked the first and second in magnitude, respectively (Figure 8B1).

## 4 Discussion

We discovered a significant association between mixed metal exposure and the levels of sNfL, a novel neurological injury marker, in the high-exposure group identified through unsupervised population stratification. This finding not only validates the effectiveness of unsupervised population stratification but also supports the link between metal exposure and the novel neurological injury marker, sNfL.

Furthermore, we found that serum sNfL levels (a marker of nerve damage) were significantly elevated in people with mixed exposures to heavy metals such as lead, cadmium, and copper, especially in men and in the general U. S. population with a body mass index greater than 25. Evidence from this study provides more valid, realistic, and generalizable findings on the neurological impacts of mixed metal exposures in the general population. Previous studies have primarily explored the effects of single metal exposure on neurological function in only a few occupation-specific populations (22). To date, no research has investigated the impact of mixed metal exposure on neurological damage in general and representative populations. Therefore, this study fills a gap in the existing literature.

Previous research on the mechanisms of metal exposure-induced nerve damage has shown that such damage is primarily through oxidative stress (28, 29) and neuroinflammatory pathways (30). For example, cadmium induces oxidative stress by altering oxidoreductase levels leading to increased reactive oxygen species (ROS) levels. This activation, in turn, stimulates the JNK and Erk1/2 pathways, resulting in neuronal apoptosis, particularly in PC12 and SH-SY5Y cells (31, 32). Cadmium enters into neurons through voltage-gated calcium channels, leading to Syn accumulation, increasing the levels of pro-inflammatory cytokines (IL-6 and TNF-α), decreasing the levels of the anti-inflammatory cytokine IL-10, and finally leading to neuroinflammation. These events lead to neuronal cell damage and degeneration (33, 34).

The toxicity of metallic lead also arises from oxidative stress (35, 36). High levels of lead cause the production of reactive oxygen and nitrogen species and deplete antioxidants (37). As lead binds directly to glutathione reductase, which contains thiol groups, glutathione—the primary antioxidant in the human body-is significantly reduced, thereby indirectly accelerating oxidative stress, eventually leading to neuronal damage and degeneration (38). Manganese acts as a transition metal in redox reactions and induces neuronal cell damage through nitrosylation of PINK1-S, inhibiting mitochondrial production by ZNF746, resulting in mitochondrial dysfunction (39, 40). Therefore, metals may contribute to neuronal damage through these pathways, leading to an elevation in the levels of sNfL.

Generally, individuals are often exposed to multiple metals in the environment rather than a single metal. We utilized BKMR and QGC to evaluate the impact of mixed metal exposure on sNfL levels in the general US population. In the BKMR, there was a significant positive correlation between co-exposure to the seven blood metals and sNfL levels in the highly exposed group, and subgroup analysis also revealed positive trends in males and overweight. Mixed exposures are a more accurate reflection of the reality of population exposure patterns. For instance, a cohort study of adult males in North China, which explored the effects of multiple heavy metal co-exposures, revealed that exposure to a mixture of seven essential metals, including calcium, copper, iron, magnesium, manganese, selenium, and zinc, in the blood was associated with a greater risk of NAFLD compared to exposure to a single metal (41). Thus, our analysis of mixed exposure to metal–metal in high and low exposure groups, following grouping by unsupervised clustering, is a more realistic analytical approach.

Numerous studies have provided evidence that metal-induced damage is more pronounced in males, primarily as a result of work-related factors. For instance, Wang et al. (42) conducted a study on male mice and found that exposure to cadmium impaired hippocampus-dependent learning and memory. Furthermore, a retrospective cohort study involving miners from Ontario, Canada, demonstrated that male miners exposed to respirable metal dust had an elevated risk of developing neurodegenerative diseases (43). Additionally, a study by Rundong Liu revealed that combined exposure to lead and a high-fat diet exacerbated cognitive decline through the CREB-BDNF signaling pathway in male rats (42), which aligns with our findings and adds weight to our conclusions.

In the subgroup of individuals with a BMI > 25, there was a significant positive correlation between metal exposure and sNfL levels. Animal experiments that exposure adult male Sprague–Dawley rats to high doses of Cd demonstrated substantial accumulation of the metal in adipose tissue (AT), particularly in subcutaneous AT (SUB-AT) (44), suggesting that overweight individuals may be at higher risk of nerve damage caused by metal exposure. This is because Cd can accumulate in adipose tissue and subsequently enter the bloodstream, where it may produce harmful effects on the nervous system (45). These findings suggest that individuals with higher BMI may have an increased vulnerability to the neurological effects of metal exposure due to the accumulation of metals in adipose tissue. However, further research is needed to fully understand the mechanisms underlying this association and to determine the specific impacts of metal exposure on neurological health in overweight individuals.

The QGC model also revealed a significant positive relationship between mixed metal exposures and sNfL levels, with zinc having the highest negative weight, highlighting the protective effect of elevated zinc levels on neurological damage (46). In contrast, cadmium and sNfL levels exhibited the highest positive consequences, which were consistent with the results of BKMR and multiple linear regression models. These observations demonstrate the robustness and validity of our study.

This study has several noteworthy limitations. Firstly, the cross-sectional design inherently limits our ability to establish causal inferences between metal exposures and sNfL dynamics. Future research should employ prospective longitudinal cohorts with repeated biomonitoring to delineate the temporal relationships and dose–response trajectories. Secondly, our analysis relied on U. S. national survey data (NHANES 2013–2014), which currently provides the only publicly available sNfL measurements. While we will incorporate updated data upon their release in future investigations, this temporal constraint limits generalizability to contemporary exposure patterns. Thirdly, single-timepoint blood metal measurements may inadequately reflect cumulative exposure burden. Future work should incorporate multi-matrix biomarkers (e.g., urinary metals, toenail biomarkers) to better characterize long-term exposure profiles. Fourthly, although we adjusted for major confounders, residual confounders from unmeasured factors (e.g., dietary zinc intake, genetic polymorphisms in metal transporters) remains possible. Future studies should incorporate comprehensive nutritional assessments and pharmacogenetic analyses to address these potential biases. Finally, the unsupervised clustering analysis relies on machine-learning algorithms, and the specific analysis mechanism is closely related to the distribution of the data, which may cause some bias, and a combination of methods is needed to subsequently validate the results.

These methodological and empirical breakthroughs yield two practical applications: (1) biomarker-driven surveillance: The established sNfL response thresholds (0.15–0.20 ln [pg/mL] per μg/L Cd increase) provide quantitative biomarkers for monitoring preclinical neurological damage in occupational health surveillance programs, and (2) precision intervention: The identified susceptibility factors (sex-specific metabolism and adiposity-mediated metal retention) enable precision prevention strategies targeting high-risk subgroups. Collectively, our findings bridge critical knowledge gaps between environmental epidemiology and clinical neurology by mechanistically linking metal exposure patterns to quantifiable neural injury biomarkers, while establishing a reproducible paradigm for investigating complex mixture effects in population health studies.

Building upon these findings, several critical research priorities emerge. First, prospective cohort studies incorporating repeated biomonitoring are imperative to establish temporal causality between metal exposure and sNfL, particularly in high-risk subgroups such as men and individuals with obesity. Furthermore, our findings in men and overweight populations suggest occupational exposure and adipose tissue-mediated metal retention mechanisms, future studies should explore these pathways through *in vitro* models and tissue-targeted metabolomics. Second, integration of multi-omics approaches (e.g., metabolomics and epigenomics) may reveal the biological mechanistic pathways linking cadmium exposure to axonal damage. Third, expanding exposure assessment to include urine metals and specimen analysis will improve the accuracy of risk characterization. Finally, targeted interventions (e.g., zinc supplementation trials) and more stringent regulatory policies for industrial emissions should be prioritized to reduce neurotoxicity risk. Future collaboration between epidemiology, toxicology, and data science needs to be strengthened, which is critical for translating these findings into actionable public health strategies.

## 5 Conclusion

This study presents the first integration of population stratification through co-exposure modeling with advanced mixture analysis, yielding three critical advances in environmental health research. First, our innovative methodology overcomes the limitations of conventional single-pollutant models by capturing complex real-world exposure patterns, with the combined application of unsupervised clustering and traditional mixture analysis representing a novel methodological contribution to exposure science. Second, the identification of high-risk demographic clusters - particularly males and individuals with obesity - provides epidemiologically validated targets for precision public health interventions. Third, we established cadmium and lead as priority hazardous metals for neurological damage, with mechanistic implications extending beyond established toxicity pathways. These findings enable evidence-based prioritization of regulatory monitoring frameworks and clinical surveillance protocols, offering a translational roadmap for detecting subclinical neurological effects and implementing primary prevention strategies in vulnerable populations. The combined methodological and substantive advances significantly enhance our capacity to address the growing global challenge of mixed metal exposures.

## Data Availability

The original contributions presented in the study are included in the article/Supplementary material, further inquiries can be directed to the corresponding authors.
