# Supplementary material for: The relationship between mixed exposure to blood metal and serum neurofilament light chain levels in the general U.S. population: an unsupervised clustering approach
Source: Front Public Health. 2025 Jul 30;13:1516879. doi: 10.3389/fpubh.2025.1516879 (PMC12343511; doi:10.3389/fpubh.2025.1516879)
Supplement: Supplementary file 1 [file Data_Sheet_1.docx]

Supplementary Material

**Table S1** Distribution of blood metal, NHANES 2013–2014.

**Table S2** Posterior inclusion probabilities (PIPs) of BKMR model

**Table S3** Association between seven blood metals and sNfL among U.S. adults by sex groups

**Table S4** Association between seven blood metals and sNfL among U.S. adults by BMI groups

**Table S5** Association between seven blood metals and sNfL among U.S. adults by age groups

**Figure S1** Spearman correlation coefficients among seven blood metals

**Figure S2** Bivariate exposure-response function plots(Generated using the BKMR software package, which describes the relationship between potential pairwise interactions between a metal metabolite (at its 25th, 50th, and 75th percentiles) and other metals fixed at its 50th percentile.)

**Figure S3** Bivariate exposure-response function plots(Generated using the BKMR software package, which describes the relationship between potential pairwise interactions between a metal metabolite (at its 25th, 50th, and 75th percentiles) and other metals fixed at its 50th percentile.)

**Table S1** Distribution of blood metal, NHANES 2013–2014.

| Metal | Detection frequency | Geometric mean | Mean | Percentile | | | | |
| --- | --- | --- | --- | --- | --- | --- | --- | --- |
|  |  |  |  | 10th | 25th | 50th | 75th | 90th |
| Blood Pb | 99.3 | 1.31 | 0.99 | 71.20 | 78.40 | 86.20 | 95.53 | 106.93 |
| Blood Cd | 91.56 | 0.50 | 0.29 | 0.12 | 0.18 | 0.29 | 0.6 | 1.17 |
| Blood Hg  Blood Mn | 91.07  100 | 1.46  9.96 | 0.85  9.41 | 0.2  6.26 | 0.44  7.61 | 0.85  9.41 | 1.56  11.8 | 3.11  14.28 |
| Blood Se | 100 | 198.24 | 196.56 | 171.49 | 183.48 | 196.56 | 210.11 | 227.08 |
| Serum Cu | 100 | 120.21 | 116.60 | 88.1 | 99.52 | 116.61 | 134.20 | 196.56 |
| Serum Zn | 100 | 88.00 | 86.31 | 71.20 | 78.40 | 86.31 | 95.50 | 106.32 |

**Table S2** Posterior inclusion probabilities (PIPs) of BKMR model

| Metal | Total | | |
| --- | --- | --- | --- |
|  | Group | Group PIP | Cond PIP |
| Blood Pb | 1 | 0.9330 | 0.8904 |
| Blood Cd | 2 | 0.7094 | 1.0000 |
| Blood Hg | 1 | 0.9330 | 0.0015 |
| Blood Mn | 1 | 0.9330 | 0.0398 |
| Blood Se | 1 | 0.9330 | 0.0244 |
| Serum Cu | 1 | 0.9330 | 0.0173 |
| Serum Zn | 1 | 0.9330 | 0.0263 |

Models were adjusted for age group, sex, race, marital status, BMI index, smoking, drinking, exercise, education level, marital status, ratio of family income to poverty, urine creatinine, hypertension, depression, cardiovascular disease, and hyperuricemia.

**Table S3** Association between seven blood metals and sNfL among U.S. adults by sex

| Metals | Female | | male | |
| --- | --- | --- | --- | --- |
|  | Coef(95%CI) | *P* value | Coef(95%CI) | *P* value |
| Blood Pb | 0.092(-0.00,0.19) | 0.073 | -0.03(-0.13,0.06) | 0.487 |
| Blood Cd | 0.141(0.02,0.26) | **0.021*** | 0.10(-0.00,0.20) | 0.052 |
| Blood Hg | 0.034(-0.05,0.12) | 0.437 | 0.03(-0.05,0.13) | 0.465 |
| Blood Mn | -0.030(-0.12,0.06) | 0.511 | 0.04(-0.05,0.13) | 0.914 |
| Blood Se | -0.043(-0.13,0.04) | 0.328 | 0.00(-0.85,0.09) | 0.435 |
| Serum Cu | 0.064(-0.04,0.17) | 0.235 | 0.89(-0.02,0.20) | 0.116 |
| Serum Zn | 0.051(-0.04,0.14) | 0.257 | -0.11(-0.19,0.02) | **0.014*** |

Models were adjusted for age group, sex, race, marital status, BMI index, smoking, drinking, exercise, education level, marital status, ratio of family income to poverty, urine creatinine, hypertension, depression, cardiovascular disease, and hyperuricemia. * *P* < 0.05; ** *P* < 0.01; *** *P* < 0.001.

**Table S4** Association between seven blood metals and sNfL among U.S. adults by BMI

| Metal | BMI<25 | | BMI>=25 | |
| --- | --- | --- | --- | --- |
|  | Coef(95%CI) | *P* value | Coef(95%CI) | *P* value |
| Blood Pb | -0.000(-0.12,0.12) | 0.990 | 0.064(-0.02,0.15) | 0.146 |
| Blood Cd | 0.061(-0.09,0.21) | 0.429 | 0.120(0.30,0.21) | **0.009**** |
| Blood Hg | 0.056(-0.05,0.16) | 0.324 | 0.011(-0.06,0.09) | 0.771 |
| Blood Mn | -0.127(-0.13,0.11) | 0.839 | 0.018(-0.06,0.09) | 0.640 |
| Blood Se | 0.012(-0.09,0.12) | 0.821 | -0.022(-0.09,0.05) | 0.575 |
| Serum Cu | 0.052(-0.07,0.18) | 0.426 | 0.053(-0.04,0.14) | 0.275 |
| Serum Zn | -0.069(-0.17,0.04) | 0.212 | -0.017(-0.09,0.06) | 0.655 |

Models were adjusted for age group, sex, race, marital status, BMI index, smoking, drinking, exercise, education level, marital status, ratio of family income to poverty, urine creatinine, hypertension, depression, cardiovascular disease, and hyperuricemia. * *P* < 0.05; ** *P* < 0.01; *** *P* < 0.001.

**Table S5** Association between seven blood metals and sNfL among U.S. adults by age

| Metal | Age>=60 | | Age<60 | |
| --- | --- | --- | --- | --- |
|  | Coef(95%CI) | *P* value | Coef(95%CI) | *P* value |
| Blood Pb | 0.052(-0.03,0.13) | 0.215 | -0.007(-0.14,0.13) | 0.921 |
| Blood Cd | 0.056(-0.03,0.14) | 0.215 | 0.293(0.14,0.45) | **0.000***** |
| Blood Hg | 0.043(-0.03,0.11) | 0.258 | 0.030(-0.09,0.15) | 0.617 |
| Blood Mn | 0.011(-0.66,0.08) | 0.775 | 0.002(-0.12,0.13) | 0.973 |
| Blood Se | -0.053(-0.12,0.02) | 0.160 | 0.060(-0.05,0.18) | 0.316 |
| Serum Cu | 0.080(-0.00,0.17 | 0.079 | 0.070(-0.06,0.20) | 0.315 |
| Serum Zn | -0.022(-0.09,0.05) | 0.548 | -0.041(-0.15,0.07) | 0.487 |

Models were adjusted for age group, sex, race, marital status, BMI index, smoking, drinking, exercise, education level, marital status, ratio of family income to poverty, urine creatinine, hypertension, depression, cardiovascular disease, and hyperuricemia. * *P* < 0.05; ** *P* < 0.01; *** *P* < 0.001.


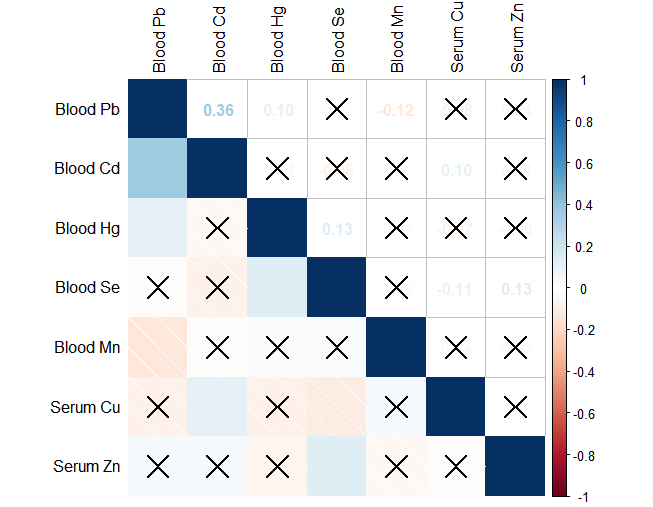


**Figure S1** Spearman correlation coefficients among seven blood metals


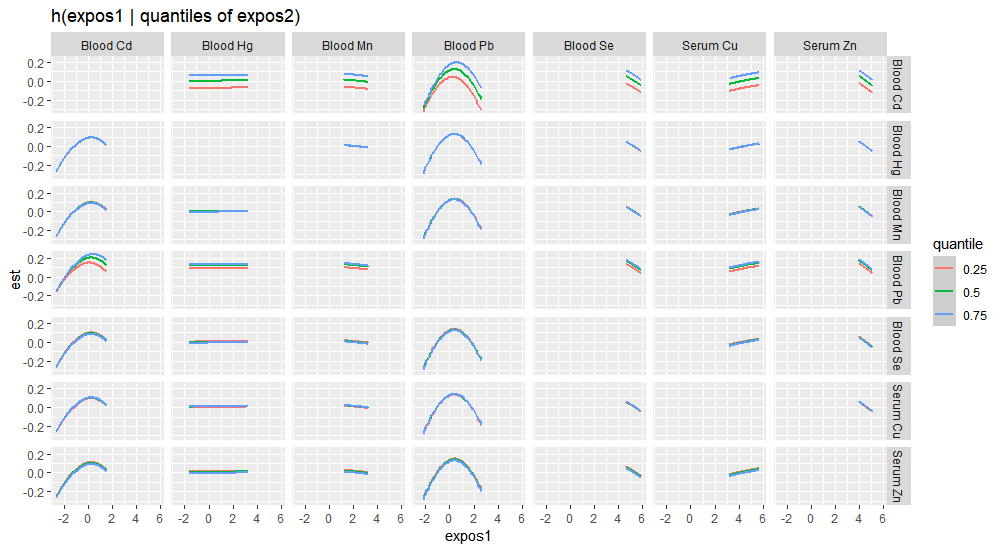


**Figure S2** Bivariate exposure-response function plots(Generated using the BKMR software package, which describes the relationship between potential pairwise interactions between a metal metabolite (at its 25th, 50th, and 75th percentiles) and other metals fixed at its 50th percentile.)


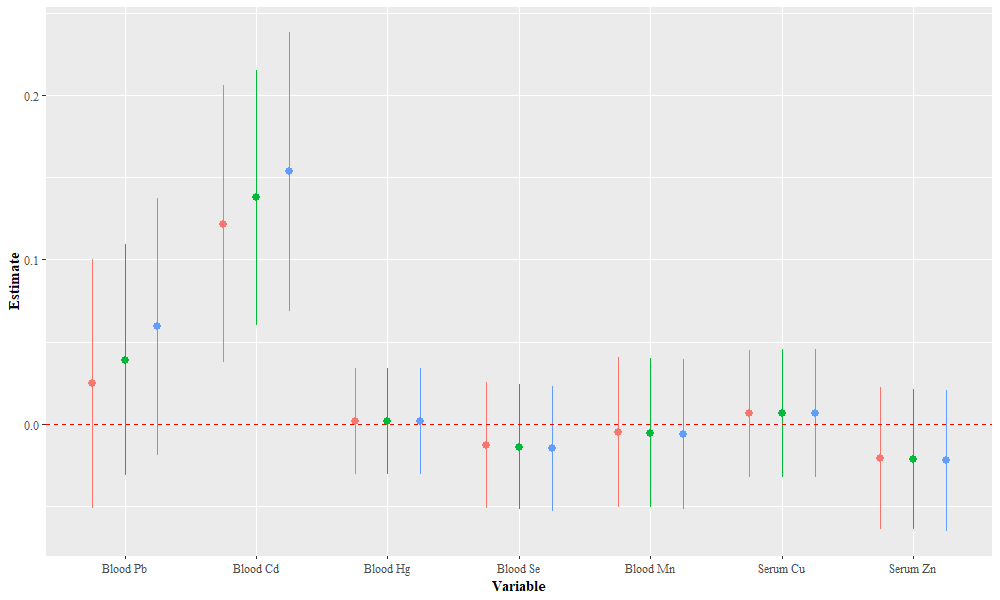


**Figure S3** The single-exposure effect of individual metals on the sNfL levels (The estimated values and 95% confidence intervals for all other metals were fixed at the 25th, 50th, or 75th percentiles by BKMR)
